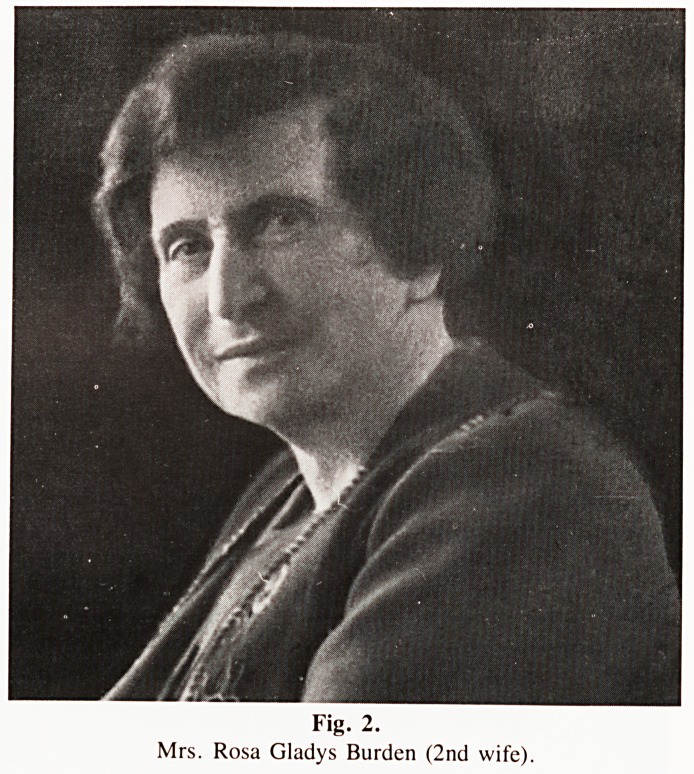# The Burdens and Bristol

**Published:** 1991-12

**Authors:** J. Jancar


### The Burdens and Bristol

**J. Jancar, MB, BCh, BAO, DPM, FRCPsych**

Rarely in a lifetime do three people achieve and contribute to society in so many ways as did the Burdens in Bristol. Here are short biographical notes and some of their major achievements.

**Reverend Harold Nelson Burden** *(1859-1930)*

From his early youth Mr. Burden apparently determined to devote his life to the welfare of others, and thus decided against the wishes of his family, to enter the Church. Like many others who had taken "the mould of man's fortune in his own hands", Mr. Burden had to enter his chosen profession without much of that material assistance his family could have so easily afforded. College life at Cambridge was thus largely denied him and he had to content himself with the simpler theological foundation then known as Ayerst Hostel. After his ordination at Carlisle in 1888, Mr. Burden spent some time in curacies in the East End of London, where he first came in contact with those unfortunates whom it was his constant ambition to help and cheer on their otherwise drab walk through life. Those early contacts undoubtedly moulded much of Mr. Burden's later activities.

Then came missionary work in Canada amongst the Ojibway Indians and the rough lumbermen of Canada's backwoods, in isolation and terrible loneliness. Here Mr. Burden experienced many vicissitudes, and "the slings and arrows of outrageous fortune" did not altogether pass him by, for he suffered severe domestic losses. In his book "Life in Algoma", published in 1984, and descriptive of his life and early struggles in the lone lands of the outposts of the British Empire. During those years in Canada he built four substantial churches and two parsonages, all of which he left entirely free from debt, besides doing his missionary work which entailed numerous journeys sometimes involving days of travel by land and water. Constant fatigue, recurrent illness from the climate with its extremes of heat and cold impaired Mr. Burden's health so that he was compelled to leave Canada. Two children had been born and had died in their infancy in Canada. He and his wife began their preparations to give up their charge and returned to England at the close of 1891. For the next two years he was Curate of Holy Trinity, Shoreditch and in 1893 entered St. Catherine's College, but being married, lived at Swavesey. Meanwhile he accepted a curacy at Milton, Cambridgeshire and acted occasionally as Assistant Chaplain at St. Catherines.

In 1895 Mr. Burden was appointed Chaplain of Horfield Prison in Bristol. About the year 1902 Mr. Burden founded the Incorporation known as the National Institutions for Persons requiring Care and Control, and became the first Warden. He

**Fig. 1.**

Rev. Harold Nelson Burden.

characteristically surrounded himself with a committee who might share in the credit of an undertaking, financed entirely by himself, a fact not then known to the public, and even now insufficiently realised. His labours were acts of faith and it was enough for him to feel he was doing his Master's work. Methodical and businesslike in his management, he had a brain for finance and was consequently enabled to administer the huge undertaking into which his modest beginnings grew, with efficiency, common sense and a commensurate economy. In 1904 Mr. Burden was appointed by the Government of the day a member of the Royal Commission then inquiring into the care of the feeble minded. This inquiry, coupled with the Commission's visits to Germany and other continental countries, in which Mr. Burden took part, so aroused his interest in the problem that he determined to devote the remainder of his life, time, mental energies, great powers to organisation and financial resources to the care and welfare of those mental unfortunates, and he continued to do so till the day of his death in 1930. With the passing of the Mental Deficiency Act of 1913 the demand for accommodation of the mentally defective became pressing, and it was partly met by Mr. Burden's activities. In fact, without Mr. Burden's help during the War years, the Board of Control might have found some difficulty in administating the Act of which it had now become the nation's official trustee.

Mr. Burden commenced by acquiring the Stoke Park Dower House from the Duke of Beaufort, then followed Heath House, Grove Beech House, Hanham Hall, Leigh Court and Whittington Hall, Chesterfield, and these he had altered and adapted — again at his own expense — to the use and requirements of mentally defective children. New blocks and houses were built and additions made to the older properties, Stoke Park itself becoming the nucleus of the group of Institutions known as Stoke Park Colony, and this, it is of historical interest to note, was the first in the British Isles to be certified under the Act of 1913 as an Institution for mental defectives.

It was no light or trivial task to found, equip, and build up from the old Dower House with a single patient an Institution, then actually housing over 1700 patients, yet this is what Mr. Burden accomplished. He had long had it in mind, when the progress of his Institutions justified it, to submit the material he had so laboriously collected to scientific analysis and investigation, and in 1927 the opportunity presented itself and was eagerly seized. His old friend, the late Dr. R. W. Branthwaite, having just retired from the Board of Control, was offered, and accepted, the position of Director of Medical Services at Stoke Park, and did so with a view to commencing research work on the scale desired by Mr. Burden. The splendid laboratories, known at Stoke Park as "the Clinic", were built and very liberally equipped. No sooner were these new activities commenced than the death of Dr. Branthwaite brought them to a temporary cessation.

Six months later Professor R. J. A. Berry was appointed to the vacant position, and it is characteristic of Mr. Burden's energetic keenness that he immediately called for a report and a scheme for the further development of research work at Stoke Park. Almost the last act of Mr. Burden's life was to approve of this report, to authorise the various medical and research appointments recommended, and to increase the financial provision far beyond the more modest scale suggested. He did not live to see his schemes for the advancement of scientific knowledge of mental disorders in actual operation, but his widow and successor in the Wardenship made it a labour of love to carry out all those of which he approved and had so much at heart. Mrs. Burden herself still further endowed mental research by a gift to the nation of £10,000.

The Clinic at Stoke Park, still further enlarged by Mrs. Burden, was one of the few laboratories in the world solely and specially devoted to the study of mental deficiency. It was well equipped, adequately financed, and liberally maintained, and one of its special features was a teaching museum of the neurological factors underlying mental deficiency. Stoke Park has thus been added, by the Senate of the University of London, to the list of Institutions recognised for practice in connection with Diploma in Psychological Medicine for candidates offering mental deficiency as their special branch, whilst the University of Bristol granted similar recognition and availed itself of the unrivalled clinical material for postgraduate study. Before his death on the 15th May, 1930, Mr. Burden had the further satisfaction of seeing the Institutions which he had founded with accommodation for over 2000 patients, put in trust for the nation for charitable purposes.

As a Freeman of the City of London and Liveryman of the Worshipful Company of Barbers, he became Master of the Barber's Company from 1924 to 1926.

**Mrs. Katherine Mary Burden** *(died 1919)*

The life of Katherine Mary Burden may be said to have been spent wholly in the service of others.

After she had completed the ordinary education for gentlewomen usual at that period, she went through a course of special training to enable her to assist others to make themselves efficient in some of the handicrafts of that time. In 1869 or early in 1870, she joined Miss Octavia Hill as assistant in the great work which she had in hand and which made that lady's name a household word throughout the country. In the next decade, whilst still almost a girl, an opportunity for special work in the East End of London was offered, and she decided to take it up. Miss Octavia Hill wrote on her leaving work with her and said:

"By her quiet, gentle manner and familiarity with the poor and their ways, and from being firm, kindly and chatty, she has been more help to me than any assistant has been for many a long day. She has all the powers that I have not, and has filled my deficiences in a way that made me look forward to working with her very much. Difficulties vanished at her touch; she always had time to chat with people, knew all the little news which throws so much light on character, noticed small excellences or neglect, kept much of the detail right, leaving me free for deeper personal intercourse with the people that I

happen to get to know best, and to meet the greater difficulties of some of their lives. I shall miss her sorely. I am glad to give her to the East. It is right she goes, and that is enough."

The characteristics noticed by Miss Octavia Hill remained with her all her life, and, coupled with her strong religious convictions, her firm faith and her hatred of anything tending to laxity of the moral code, proved of the utmost value in much that she had to do. Her work in the East End of London covered a period of some sixteen years and influenced the lives of thousands of persons, the greater part of whom were work girls whose welfare she made her special study and care. Among many other things, she personally supervised social clubs established for their social and spiritual good. During this period she was generally known in East London as "Miss Kate", and many were the happy wives and mothers, who, but for her, would have become fallen women and never known either of these happy states.

This work ended on her marriage to the Rev. Harold Nelson Burden. She went with her husband to Canada and was his constant comfort and support during three years' strenuous work. In that period, in addition to the regular spiritual care of the people scattered over nearly 300 square miles, churches were erected, graveyards made and other provisions carried out for the better administration of the Church's work in the district, in all of which she did her full part.

On their return to England, the East End again had their sympathetic assistance for a time. In 1895 they moved to Bristol and were instrumental in the promotion and erection of the Royal Victoria Home. The Home was at first intended for the care of inebriate women and of girls in danger of falling, but before it was completed a suggestion came from the Home Office that the work might helpfully also include the care of women convicts whose crimes and history before and after conviction showed them to be suitable for the clemency of the Secretary of State. The suggestion was considered a proper one to adopt and a wing was added for their reception.

Mrs. Burden took a leading part in the work, aided it financially and brought her personal influence to bear on the inmates with much success.

The passing of the Inebriates Act of 1898 made much larger accommodation for inebriates necessary. Premises were taken at Brentry and the assistance of County and County Borough Councils invited, 24 of whom decided to contribute to the establishment of the institution. For three years both Mrs. Burden and her husband gave it their continuous attention. Every woman who entered the institution came under Mrs. Burden's influence and received her help.

In the early part of 1903, the greater need of the National Institutions became clear to them, and the Institutions for Inebriates at Ackworth, Chesterfield, Harling and Lewes had the care hitherto restricted to Brentry. A few years later work for the mentally defective was added to the existing work of the National Institutions. Mrs. Burden's labour of love was extended to them. She constantly visited the whole of the Institutions of the Incorporation of National Institutions and each year travelled many thousands of miles in so doing. Her influence on the respective staffs and inmates never failed. The work during the fourteen years from 1903 four times doubled itself, but up to the day on which her work ceased for ever, her visits to Institutions were continued with unfailing regularity.

Stoke Park Colony, with its six ancillary Institutions, was the largest, not only of the National Institutions, but of all Certified Institutions in the Country. A large part of Mrs. Burden's time was spent at the Colony. Her bright, kindly winning presence was a constant pleasure to the staff, and delight to the patients, especially to the little ones, of whom she was particularly fond.

She died on 25th October, 1919 and is buried at Ridgeway Park in Bristol, the usual place of burial for patients passing away at Stoke Park Colony.

**Mrs. Rosa Gladys Burden** *(1891-1939)*

After the death of his first wife, Mr. Burden married Miss R. G. Williams, who was the Superintendent of Stoke Park. Following the death of her husband, Mrs. Burden was appointed, in 1930, Warden at Stoke Park Colony.

**Fig. 2.**
Mrs. Rosa Gladys Burden (2nd wife).

In 1933 she donated the sum of £10,000 and with the gift expressed her desire that it should primarily be devoted to problems underlying the causation and inheritance of normal and abnormal mentality. The Burden Mental Research came into being.

In 1935 Mrs. Burden, as the Chairman of the Trustee of the Burden Trust, built at Stoke Park, at the suggestion of a Surgeon, a clinic for surgical treatment of the mentally defective patients in Stoke Park Colony. The idea was later abandoned and the Medical Research Council suggested that the premises be used as a neuro-research centre for the West of England. Mrs. Burden accepted the idea and gave further financial support and the Burden Neurological Institute was opened on 12th May, 1939. Professor F. L. Golla was appointed Director of the Institute, Dr. W. Grey Walter was in charge of the Physiological Research Unit, Dr. E. L. Hutton was in charge of Psychiatric Research, Mr. L. D. MacLeod and Mr. A. Tingey were appointed as biochemists and Professor Max Reiss was in charge of the Endocrinological unit.

Sir Wylie McKissock, Mr. Willway and Professor Lambert Rogers, gave their services in the beginning, later the Surgeon-in-Charge was Miss Diane Beck. These neuro-surgeons and the staff dealt with all the neuro-surgical casualties from the West Country for several years.

The Institute became nationally and internationally known for its work, especially in the studies of electro-physiology of the C.N.S., electro-encephalography, and the physical treatment of mental disorder. Patients at the Stoke Park Group are benefiting greatly from all the latest advances at the Burden Institute. Just before the Second World War, the Burden Institute initiated the practice of electric convulsive therapy in this country, and soon after the first leucotomy in Great Britain was performed there. Since then many discoveries and observations have been made in the Institute.

The Burden Trustees are still financing research projects in the United Kingdom, and in addition they are generously supporting the Burden Institute.

In 1969 the Burden Trustees instituted "The Burden Research Gold Medal and Prize" to encourage research into mental handicap. To date 11 eminent Psychiatrists in this field have received this coveted award.

## Figures and Tables

**Fig. 1. f1:**
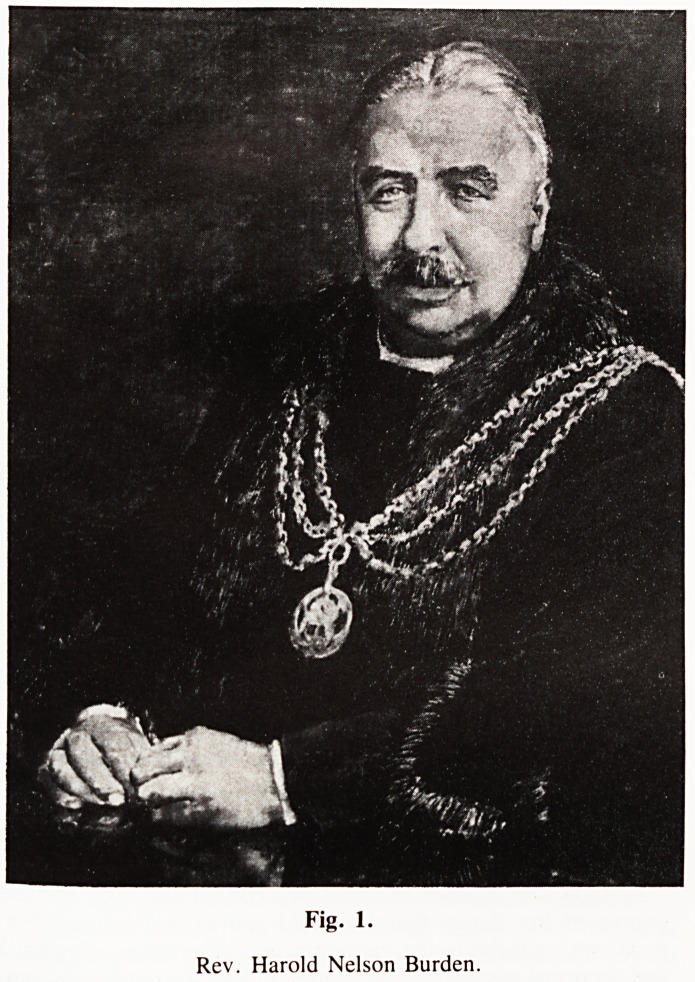


**Fig. 2. f2:**